# Perceptual illusion of body-ownership within an immersive realistic environment enhances memory accuracy and re-experiencing

**DOI:** 10.1016/j.isci.2021.103584

**Published:** 2021-12-08

**Authors:** Heather Iriye, H. Henrik Ehrsson

**Affiliations:** 1 Department of Neuroscience, Karolinska Institutet, Stockholm 171 77, Sweden

**Keywords:** Memory structure, Machine perception

## Abstract

Our bodies provide a necessary scaffold for memories of past events. Yet, we are just beginning to understand how feelings of one's own body during the encoding of realistic events shape memory. Participants formed memories for immersive, lifelike events by watching pre-recorded 3D videos that involved a first-person view of a mannequin's body through head mounted displays. We manipulated feelings of body ownership over the mannequin using a perceptual full-body illusion. Participants completed cued recall questions and subjective ratings (i.e., degree of reliving, emotional intensity, vividness, and belief in memory accuracy) for each video immediately following encoding and one week later. Sensing the mannequin's body as one's own during encoding enhanced the following factors: memory accuracy across testing points, immediate reliving, delayed emotional intensity, vividness, and belief in memory accuracy. These findings demonstrate that a basic sense of bodily selfhood provides a crucial foundation for the accurate reliving of the past.

## Highlights

• Body ownership during the encoding of lifelike events is core to memory retrieval
• Disrupting body ownership during encoding reduces accurate recall of event details
• Disrupting body ownership decreases reliving immediately after encoding
• Emotional intensity, vividness, and belief in memory accuracy decline after a week

## Graphical abstract

**Figure:**
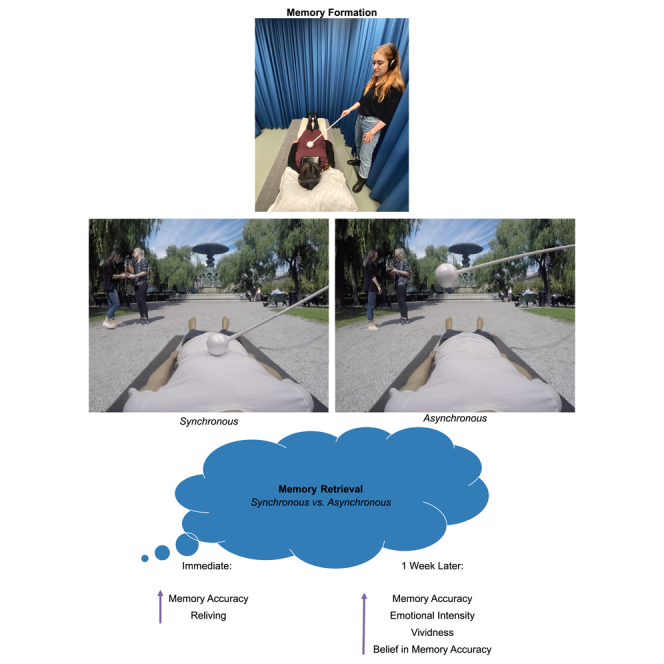

## Introduction

Memories of past experiences are inextricably linked with having a body (Damasio, 1999; Rubin, 2006; Wilson, 2002). As an event unfolds, a diverse array of sensory, motor, kinaesthetic, and affective experiences is anchored to the body, including multisensory signals that underpin feelings of body ownership, i.e., the perception of one's body as belonging to oneself (Ehrsson, 2020). The perception of one's body as one's own binds incoming multisensory signals into a unified experience as memories for events are formed. During retrieval, the same memory components related to the perception of one's body and the external environment present during encoding are reactivated (Rubin, 2006), enabling recall of specific memory content (i.e., spatial, temporal, affective, and contextual details). Critically, reactivation of disparate memory traces stored within the context of an embodied physical self is crucial to creating a subjective sense of reliving the past event (i.e., autonoetic consciousness), which is the defining feature of episodic memory (Tulving, 1985). Thus, the sense of our own body in the center of spatial experience, a fundamental layer of selfhood, plays a critical role in determining how memories of past events are formed and retrieved.

Although previous research has often highlighted the close link between memory and psychological selfhood (Brewer, 1986; Conway and Pleydell-Pearce, 2000), the role of the perceptual experience of one's own body is typically not explicitly considered. For example, according to the self-memory system model of autobiographical memory, memories of past events are a scaffold for a coherent sense of selfhood over time and selectively retrieved according to the self's current goals or motivations (Conway and Pleydell-Pearce, 2000; Conway, 2005, 2009). Further, autobiographical memories (i.e., memories of personal past events) are defined by the fact that they are self-referring (Brewer, 1986), and extensive research demonstrates that we are biased to remember self-related information (i.e., self-memory bias; Conway and Pleydell-Pearce, 2000; Cunningham et al., 2008; Sui and Humphreys, 2017; Symons and Johnson, 1997). Yet, we are only just beginning to understand how our most basic form of selfhood – the coherent perception of a single own body in the center of our spatial experience – influences memory formation and retrieval.

Body ownership can be experimentally manipulated through perceptual full-body illusions (Petkova and Ehrsson, 2008). The full-body illusion is a perceptual bodily illusion just like the rubber hand illusion (outside VR; Botvinick and Cohen, 1998; Ehrsson et al., 2004), obeys similar temporal and spatial multisensory rules, and is associated with similar activation of multisensory brain regions (Petkova and Ehrsson, 2008; Petkova et al., 2011; Ehrsson, 2020). Participants are fitted with head-mounted display units that depict a mannequin's body viewed from a first-person perspective aligned with the participant's real body. Next, a brush is used to stroke the torso of the mannequin while brushstrokes are applied to the participant's physical body either synchronously or in an alternating pattern. Synchronous visual and tactile signals applied to the participant's and mannequin's torso lead to a sense of ownership over the mannequin because of multisensory integration. When two or more signals from two or more sensory modalities are presented simultaneously in the same place, they will be integrated and perceived as a unified whole (Ehrsson, 2020). In the case of the full body illusion, the visual impression of the mannequin being stroked perceptually fuses with the somatosensory feelings from the real body, and the participant experiences ownership over just a single body (i.e., the mannequin). In contrast, asynchronous visual and tactile signals are not integrated into a single coherent multisensory representation, and they either reduce or abolish feelings of ownership over the mannequin's body. Consequently, participants' cohesive sense of bodily selfhood is disrupted because the body that they see (i.e., the mannequin) does not feel like the body that they own. Rather, vision and somatosensation are segregated, and the mannequin is experienced as an external object distinct from the real body one feels “behind” the scene presented in the head-mounted display. Critically, contrasting synchronous with asynchronous visuotactile stimulations between the mannequin and the participant's real body allowed the elicitation or elimination of multisensory integration of visual information from the mannequin and somatosensory information from the participant's real body in otherwise identical simulation conditions (i.e., the participant *never sees their real body*, but always the same mannequin).

Previous research has suggested that coherent perceptions of bodily self in space are critical for optimal memory performance, implying that we are better able to remember information if it is encountered when we have a consistent sense of our bodies to ground our experiences (Bergouignan et al., 2014; Bréchet et al., 2019, 2020; Tacikowski et al., 2020). However, these studies have either not investigated how the fundamental perception of one's body as belonging to oneself influences memory for lifelike events, and/or failed to provide clear measures of memory accuracy and key subjective properties of memory retrieval (i.e., reliving, emotional intensity, and belief in memory accuracy). For example, Bergouignan et al. (2014) focused on the impact of shifts in visual perspective and self-location on memory, rather than body ownership. Participants experienced realistic social interactions while wearing a virtual reality headset linked to a camera positioned either directly above their physical body to mimic a natural first-person perspective, or outside their body such that it was visible from a third-person perspective. The authors manipulated perceived self-location by repeatedly moving a rod just below the camera and simultaneously touching the participant's physical body at the chest (Ehrsson, 2007) before the social interaction. Importantly, the authors did not manipulate body ownership because a control condition involving asynchronous touches was not included. After a one-week delay, participants freely recalled memories for these experiences and completed remember/know judgments on specific event details. The authors found that repeated retrieval of memories encoded from an out-of-body compared to in-body perspective was associated with reduced activation of the posterior hippocampus during early retrieval attempts but increased activation of the same region during late retrieval attempts, which was interpreted as reflective of impaired binding of episodic information during encoding. The authors also found that participants were less likely to assign “remember” as opposed to “know” judgments to details of events encoded from an out-of-body perspective. Remember/know judgments were originally intended to dissociate recollection (i.e., the ability to mentally travel back in time and consciously recall the context in which information was learned) from familiarity (i.e., a feeling that an item has been encountered before but without the ability to recall the context; Tulving, 1985). However, remember/know judgments do not actually provide a clear measure of subjective reliving (Rubin and Umanath, 2015; Wais et al., 2008). Rather, evidence suggests that they indicate memory strength (i.e., “remember” responses reflect high recollection and familiarity and “know” responses reflect low recollection and familiarity; Wixted and Mickes, 2010), whereas alternate findings show that they correlate more with belief in the accuracy of a memory than sense of reliving (Rubin et al., 2003; Rubin and Siegler, 2004). In addition, remember/know judgments do not assess whether retrieved memory contents are empirically accurate or false. Thus, although Bergouignan et al. (2014) provide important insights on how visual perspective and self-location during memory encoding influence neural activity during retrieval, it does not directly address the role that a central aspect of bodily selfhood, namely the perception of our bodies as belonging to ourselves, plays in determining memory accuracy and subjective re-experiencing.

A separate set of studies investigated how recognition memory accuracy is affected by exploring static, immersive scenes with a projection of one's real body compared to without a visible body (Bréchet et al., 2019, 2020). However, similarly to Bergouignan et al. (2014), the authors did not investigate body ownership specifically and their choice of memory measure did not clearly assess subjective re-experiencing during retrieval. In these studies, participants were immersed within realistic virtual environments each containing a set of unique objects that were explored with either a real-time video of the participant's actual body seen from a first-person perspective or without a visible body. After freely exploring the virtual scene with head movements for a brief period, participants were instructed to point to a white ball that bounced around the scene to ensure that each part of the room was seen. Both immediately after environments had been encoded after a one-hour delay, and participants were re-immersed within virtual scenes and asked to indicate whether they were identical to the scenes from the initial session. Participants were more likely to correctly recognize scenes they had initially encoded with a body compared to without a body at delayed testing. These results were replicated in a subsequent study and expanded upon to show that the visual presence of one's body within a virtual scene also leads to enhanced recognition of scenes experienced before the encoding session (Bréchet et al., 2020). Yet, the influence of experiencing virtual scenes with a visible body compared to without one on body ownership is unclear. One can still feel ownership over one's body in visually degraded conditions, such as in darkness, fog, or in “invisible body illusions” (D'Angelo et al., 2017; Guterstam et al., 2015a; Kondo et al., 2018). Moreover, an effect of multisensory body representation on memory performance cannot be disentangled from a bias for self-related information simply based of the visual recognition of one's body that is being displayed in the immersive scene, because the highly self-relevant and familiar experience of viewing one's real body compared to the unfamiliar experience of invisibility. A separate issue is that even though the authors relate their findings to the “subjective feeling of re-experiencing the past events”, their recognition memory paradigm does not truly assess subjective reliving (Wheeler et al., 1997). Rather, recognition memory tasks can be performed purely based on feelings of familiarity, rather than genuine reliving (Wheeler et al., 1997), and do not assess the ability to mentally travel back in time and vividly relive past events (Tulving, 1985). Moreover, memory retrieval involves an array of phenomenological dimensions such as vividness (i.e., the clarity of mental images), emotional intensity (i.e., the strength rather than valence of emotions), and belief that the event actually occurred, which all make unique contributions to how a memory is re-experienced that were not examined.

One recent study also used a recognition memory paradigm to demonstrate that experiencing a friend's body as one's own impairs memory accuracy (Tacikowski et al., 2020). Pairs of friends were asked to rate how well a set of personality traits applied to each other. Participants were then asked to lie on a bed wearing a head mounted display unit, in which either their own body or the body of their friend lying next to them was visible. The experimenters applied synchronous or asynchronous strokes to participants' body parts. Synchronous strokes induced feelings of ownership over the visible body, whereas asynchronous strokes diminished or abolished the illusion. Next, participants rated how strongly they felt a random subset of the original list of personality traits applied to them, instead of their friend. At the end of the experiment, participants completed a surprise recognition memory task where they were presented with the personality traits rated earlier mixed in with new traits that were not previously encountered and decided whether the trait was previously seen or not. The authors found that recognition memory accuracy for personality traits rated during the friend-body-swap illusion was worse compared to the control conditions that did not induce the illusion. However, this memory impairment was mitigated if participants rated their personality traits as more similar to their friends' after experiencing the friend-body-swap illusion. Recognition memory accuracy was also reduced when participants received asynchronous strokes while viewing their own body, which led to a sense of disownership over their own bodies, compared to synchronous strokes. Together, these results suggest that disrupting default feelings of body ownership, either by embodying the body of a psychologically dissimilar friend or losing ownership over one's own body, impairs memory encoding. Yet, as with the drawbacks in the study designs of Bréchet et al. (2019, 2020), recognition memory paradigms do not assess reliving (Wheeler et al., 1997) or other key phenomenological properties of retrieval that enable a past event to be strongly re-experienced (e.g., vividness, emotional intensity, and belief in memory accuracy). Belief in memory accuracy is of particular interest in light of clinical disorders that involve both false belief in events that have not actually occurred and disrupted bodily self-representation, such as schizophrenia and dissociative disorders that characterized by depersonalization and derealization (APA, 2013; Borda and Sass, 2015). Thus, research to date does not directly assess how body ownership influences either the subjective sense of reliving of past events or objective accuracy with which specific event details are retrieved. Developing an understanding of the relationship between the perception of one's body as one's own and memory for events is important because it has the potential to optimize memory and retain the ability to vividly relive the past in the context of healthy aging, as well as clinical disorders affecting memory (e.g., Alzheimer's Disease) and disrupted body representation (e.g., depersonalization disorder).

In the present study, we aimed to investigate how body ownership during the encoding of lifelike events affects memory accuracy and subjective re-experiencing both immediately and after a one-week delay. We induced a perceptual full-body illusion to manipulate feelings of ownership over a mannequin's body visible from a first-person perspective as participants watched immersive, pre-recorded videos of lifelike events through head-mounted displays. We measured memory accuracy through cued recall questions that pertained to either central event details (i.e., information relevant to the main story in the scene) or peripheral event details (i.e., information in the background of the scene that was not relevant to the main story). We assessed memory accuracy for both types of event details to align with previous paradigms from the fields of autobiographical memory (Berntsen, 2002), eyewitness memory (Steblay, 1992; Yuille and Cutshall, 1986), event memory (Iriye and St. Jacques, 2021), episodic memory (Sekeres et al., 2016), perception (Öhman et al., 2001), and virtual reality (Gorisse et al., 2017). The subjective re-experiencing of memories was measured through ratings of reliving, vividness, emotional intensity, and perceived memory accuracy at both testing sessions.

We predicted that intact compared to disrupted feelings of ownership over the mannequin's body at encoding would lead to heightened memory accuracy and subjective re-experiencing (i.e., reliving, vividness, emotional intensity, and belief in memory accuracy) during retrieval. We did not have specific predictions about how a sense of body ownership would influence central compared to peripheral details but included both types of event details in the cued recall tests so that our paradigm was consistent with established standards in memory research. As previous studies have reported effects of various manipulations of the experience of bodily self in space on memory immediately after encoding (Tacikowski et al., 2020), at a one-hour delay (Bréchet et al., 2019, 2020), and a one-week delay (Bergouignan et al., 2014), we had no specific predictions about whether body ownership would influence objective and subjective memory components at immediate or delayed testing.

## Results

**Figure 1:**
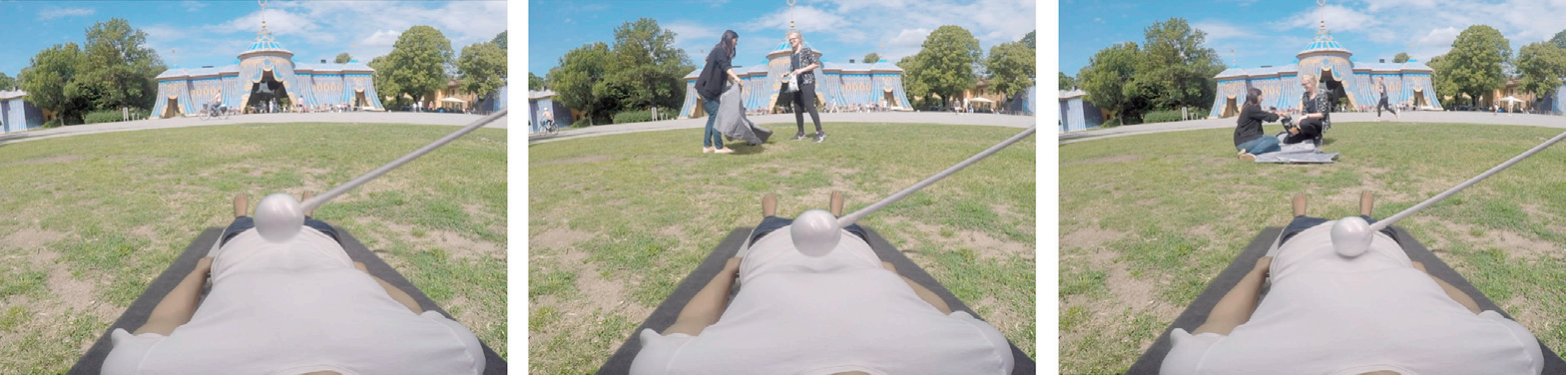
Screenshots of an example video: Hagaparken Each immersive video involved a first-person view of a mannequin's body that was continuously stroked with the Styrofoam ball as an event unfolded within the scene. Participants watched videos through an HMD and felt strokes on their actual torso either synchronously or asynchronously with the strokes in the video. Note that footage from only one eye is shown here. Actual stimuli consisted of two videos taken from slightly different positions corresponding to a left and right eye centered in the middle of the HMD to create a stereoscopic effect.

The study involved two sessions spaced one week apart (*N* = 30). During session one, we immersed participants within 24 pre-recorded videos viewed through a head mounted display, which depicted realistic, everyday events that included a natural first-person view of a mannequin's reclining body which was aligned with the location of the participants real bodies (see Figures 1 and S1). We used a full-body illusion to manipulate the multisensory perception of the mannequin's body as one's own body (Petkova and Ehrsson, 2008). Throughout each video, a white Styrofoam ball attached to a wooden stick repeatedly touched the mannequin's torso. The experimenter touched the participant's real torso either synchronously or asynchronously with the video. Synchronous touches induced an illusory sense of body ownership over the mannequin, whereas asynchronous touches significantly reduced or eliminated the illusion. Halfway through each video, two characters engaged in a brief dialogue on a unique topic.

Immediately after participants finished watching the videos, objective memory accuracy was assessed by asking participants to complete cued recall questions that pertained to each event's central details (i.e., facts pertaining to the main storyline, e.g., which animal in the museum was Heather surprised to see?) and peripheral aspects of the surrounding scene (e.g., identity of shops visible in the background). The subjective re-experiencing of memories was assessed through ratings of reliving, vividness, emotional intensity, and belief in memory accuracy. One week later, participants returned to the laboratory for session two where we first assessed the strength of the full-body illusion. Participants were immersed within two previously unseen videos, one involving synchronous and the other asynchronous visuotactile stimulation, and asked to rate three statements measuring the degree of embodiment over the mannequin and three control statements on seven-point Likert scales (−3 = Strongly Disagree, 0 = Neutral, 3 = Strongly Agree; see Table 1). Note that these new videos were only used to measure the strength of the full-body illusion and were separate from the videos used to assess memory from the first session. Next, participants completed a cued recall accuracy test with new questions pertaining to the videos encoded the previous week and the same subjective ratings made during session one. We chose a one-week interval between testing sessions as richness of recollection and sense of reliving are known to decline with a delay of this length (Tulving, 1985).

**Table 1:** Questionnaire statements

Illusion	I1	I Experienced that the touch I felt was caused by the brush touching the mannequin
	I2	I felt that the touch of the brush on the mannequin in the location where I saw the brush moving
	I3	I felt as if the mannequin I saw was my body
Control	C1	I could no longer feel my body
	C2	I felt as if I had two bodies
	C3	When I saw the brush moving, I experienced the touch on my back

### Successful induction of the full-body illusion

**Figure 2:**
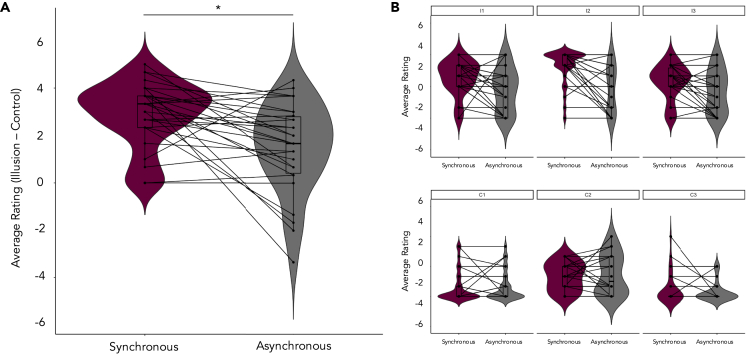
Illusion induction questionnaire ratings (A) Average illusion statement ratings were corrected for susceptibility to demand characteristics by subtracting average control statement ratings from average illusion statement ratings separately for each conditions. The synchronous condition was associated with higher ratings, indicating that participants felt a greater sense of ownership over the mannequin's body relative to the asynchronous condition. (B) Average statement ratings that assessed illusion strength (I1-I3) were consistently higher in the synchronous compared to asynchronous condition. In contrast, average ratings pertaining to control statements (C1-C3) did not differ between conditions. Plot shows means ± SE. *p* = .001.

We calculated illusion statement ratings corrected for suggestibility and response bias by subtracting average control statement ratings from average illusion statement ratings separately for each condition. Crucially, the difference between illusion and control items was greater for the synchronous condition (*M* = 2.89, *SD* = 1.40) compared to the asynchronous condition (*M* = 1.48, *SD* = 1.88), *Z* = −3.32, *p* = .001, *d* = .65, two-tailed Wilcoxon signed-rank test, see Figure 2A). In line with this, a direct comparison of the average illusion statement ratings across the two conditions was also significant, *t*(29) = 4.62, *p* < .001, *d* = .84. Importantly, average responses to each illusion statement were positive in the synchronous condition, indicating participants agreed that they felt the multisensory illusion of the mannequin's body as their own (see Figure 2B). In contrast, in the asynchronous condition, these illusion-related ratings were negative meaning that the participants on average did not affirm the illusion. The control statements received negative ratings in both conditions with no significant difference between conditions (synchronous: M = −1.80, SD = 1.04; asynchronous: M = −1.80, SD = 1.19; Z = −.14, p = .89), suggesting that suggestibility or response bias did not change based on the type of visuotactile stimulation. Collectively, these findings confirm that participants felt a stronger illusion of embodying the mannequin following synchronously compared to asynchronous visuotactile stimulation and that the illusion paradigm worked as expected.

### Memory accuracy linked to body ownership at encoding

**Figure 3:**
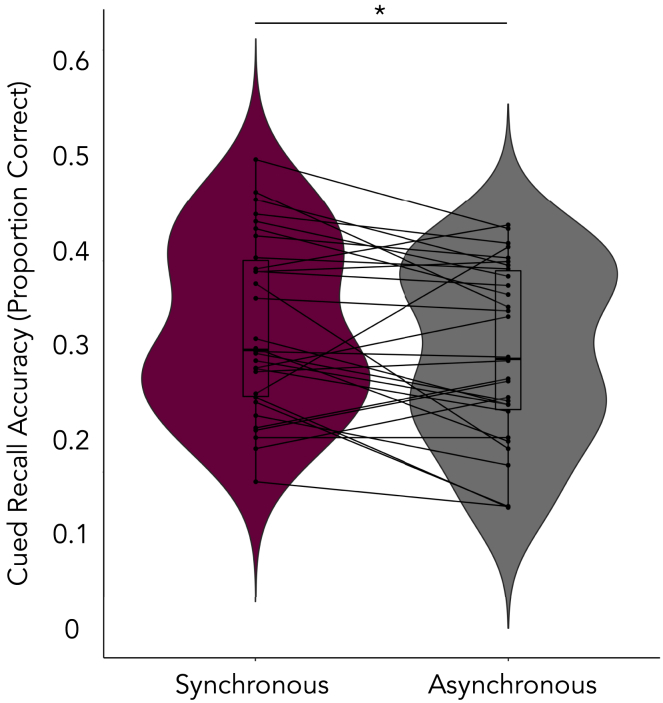
Cued recall accuracy The synchronous condition was associated with increased cued recall accuracy, relative to the asynchronous condition. Plot shows means ± SE. *p* = .03.

We conducted a 2 (Testing Session: Immediate, Delayed) x 2 (Detail: Central, Peripheral) x 2 (Visuotactile Synchrony: Synchronous, Asynchronous) repeated measures ANOVA on the percentage of correct responses to the cued recall questions. We observed a main effect of visuotactile synchrony, F(1,29) = 5.32, *p* = .03, η^2^ = .16, that indicated higher cued recall accuracy following synchronous stimulation (*M* = 36.62, *SD* = 9.15) compared to asynchronous visuotactile stimulation (*M* = 34.03, *SD* = 9.13; see Figure 3). There were further main effects of testing session, *F*(1,29) = 59.13, *p* < .001, η^2^ = .67, and detail, *F*(1,29) = 131.07, *p* < .001, η^2^ = .82, which were qualified by a two-way interaction, *F*(1,29) = 65.54, *p* < .001, η^2^ = .69. Post-hoc tests adjusted for multiple comparisons using Bonferonni corrections indicated that percentage of correct responses decreased across testing sessions for central details, *p* < .001 (Immediate: *M* = 55.62, *SD* = 13.15; Delayed: *M* = 37.11, *SD* = 13.67), but not peripheral details (Immediate: *M* = 19.53, *SD* = 8.44; Delayed: *M* = 17.87, *SD* = 8.51; see Figure S2). No other main effects or interactions were observed.

**Figure 4:**
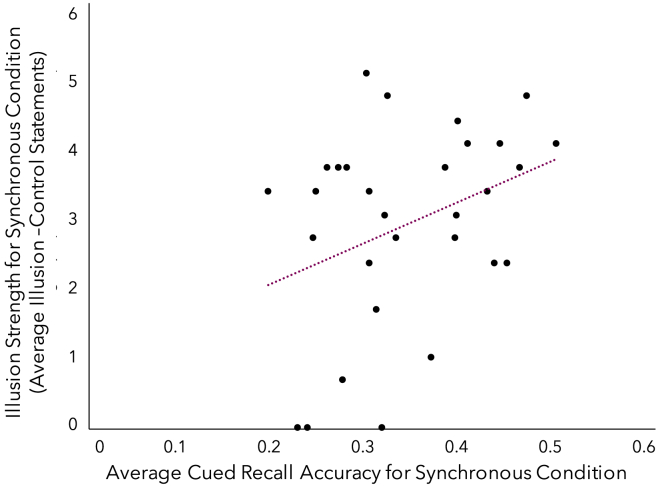
Correlation between illusion strength and cued recall accuracy in the synchronous condition A Spearman's rank order correlation found that the strength of the full body illusion was marginally positively correlated with the ability to accurately retrieve details of events encoded with synchronous visuotactile stimulation. *p* = .06.

We conducted a post-hoc Spearman's rank-order correlation to determine the relationship between the strength of the full-body ownership illusion (i.e., average illusion – average control statement ratings in the synchronous condition) and average cued recall accuracy in the synchronous condition. There was a marginally significant weak positive correlation between illusion strength (average illusion statement rating – average control statement rating) and cued recall accuracy for details pertaining to videos encoded with synchronous visuotactile stimulation averaged across testing points (*r*_*s*_(28) *=* .34, *p* = .06; see Figure 4). This finding suggests that the more participants felt the mannequin's body as their own, the better they were able to recall the specific details of the event at trend-level.

### Memory phenomenology linked to body ownership at encoding

**Figure 5:**
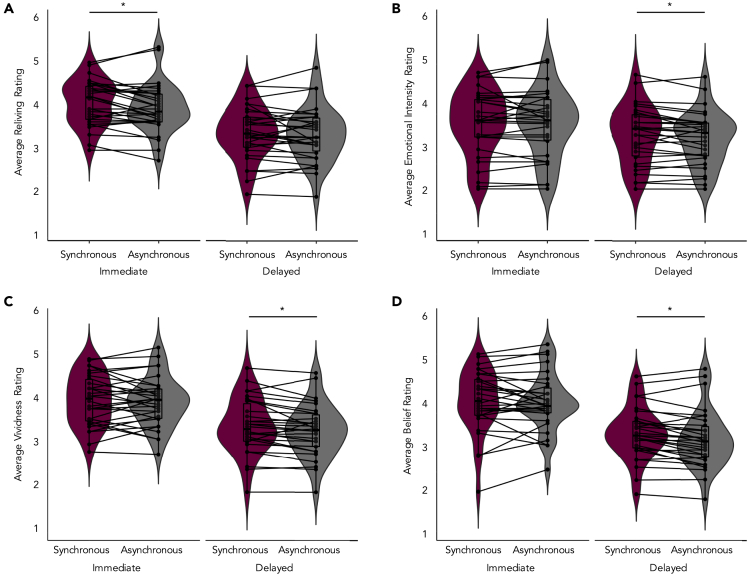
Subjective ratings (A–D) (A) Reliving ratings were higher in the synchronous compared to asynchronous conditions at immediate testing. Emotional intensity (B), vividness (C), and belief in memory accuracy, (D) were higher in the synchronous compared to asynchronous conditions at delayed testing. Plot shows means ± SE. All *p's* < .03

We conducted a 4 (Rating: Reliving, Emotional Intensity, Vividness, Belief) x 2 (Testing Session: Immediate, Delayed) x 2 (Visuotactile Synchrony: Synchronous, Asynchronous) repeated-measures ANOVA on average subjective ratings. There were main effects of rating, *F*(1.48, 42.94) = 5.76, *p* = .01, η^2^ = .17, testing session, *F*(1,29) = 38.29, *p* < .001, η^2^ = .57, and visuotactile synchrony *F*(1,29) = 6.53, *p* = .02, η^2^ = .18. Main effects were qualified by a two-way interaction between rating and testing session, *F*(2.03, 58.81) = 8.80, *p* < .001, η^2^ = .23, and a three-way interaction between rating, testing session, and visuotactile synchrony *F*(3,87) = 3.03, *p* = .03, η^2^ = .10. Post-hoc tests adjusted for multiple comparisons with Bonferroni corrections revealed that reliving ratings were higher for the synchronous compared to asynchronous condition at immediate testing, *p* = .02 (see Figure 5A). At delayed testing, the synchronous compared to asynchronous condition was associated with higher emotional intensity, *p* = .03, vividness, *p* = .007, and belief in memory accuracy, *p* = .001 (see Figures 5B–5D). Post-hoc Spearman's rank-order correlations did not reveal any significant relationships between illusion strength (i.e., average illusion – average control statement ratings in the synchronous condition) and average individual subjective recall ratings (reliving, vividness, emotional intensity, and belief) in the synchronous condition.

## Discussion

Here, we investigated how the perception of body ownership during encoding contributes to the rich and accurate reliving of past events. We show that synchronous multisensory stimulation leading to a coherent perceptual experience of a mannequin's body as one's own during the encoding of lifelike events enhances the accurate retrieval of associated narrative, visual, and contextual details (i.e., event details assessed by the cued recall tests) compared to a control condition when the mannequin was just perceived as an external object. Cued recall accuracy was higher during retrieval of events encoded with synchronous compared to asynchronous stimulation at both immediate and delayed retrieval, and this effect was predicted by the strength of the full body illusion. The present study is the first to directly investigate the influence of body ownership during encoding on subsequent reliving during retrieval. Our findings indicate that one's sense of ownership over a body embedded within a realistic scene during encoding boosts reliving immediately after an event is encoded compared to when body ownership is disrupted, but that this effect fades over time and is no longer present one week later. Further, illusory feelings of ownership over the mannequin as the event unfolded were associated with increased vividness, emotional intensity, and perceived memory accuracy at delayed retrieval. Together, the results suggest that a fundamental sense of a coherent multisensory physical self, ties visual, spatial, emotional, and contextual features of an event together as it unfolds, allowing it to be re-experienced in the future. Specifically, the perceived own body defines the center of ego-centric space, which is the coordinate system in which sensory, emotional, and other cues are coded spatially with respect to the self (Ehrsson, 2020). Without a fundamental sense of body ownership to ground our experiences in a single body within a coherent spatial world, both objective and subjective memory properties are lost over time.

We built upon previous research to uncover new insights into how own-body perception during memory formation influences the accuracy of retrieval. Prior studies have demonstrated that recognition memory accuracy is impaired following disruptions to feelings of body ownership, either by inducing the friend-body-swap illusion or disownership of one's seen real body through asynchronous visuotactile stimulation (Tacikowski et al., 2020); in addition, similar memory effects were observed when exploring scenes with one's own visible body compared to an invisible body (Bréchet et al., 2019, 2020). However, memories for events in real life are more complex than memorizing a list of words and rarely involve viewing a static room. Rather, memories “in the wild” involve other people dynamically engaged in conversations and interacting with the world around them. Although Bergouignan et al. (2014) more closely recreated real-life experiences, they did not isolate the specific contribution of body ownership to memory. Instead, they used a more complex out-of-body illusion to perturb the conscious self-experience involving a combination of changes in visual perspective from which the real body is viewed (Ehrsson, 2007), loss of ownership of the real body (Guterstam and Ehrsson, 2012; Guterstam et al., 2015b), and changes in self-location to an illusory location within the scene (Guterstam and Ehrsson, 2012; Guterstam et al., 2015b). Moreover, the remember/know task employed did not provide a clear measure of memory accuracy. To directly assess how the perception of owning a body in the scene impacts accuracy for details of lifelike events, we asked participants in the present study to answer cued recall questions about immersive experiences that involved a storyline between two main characters situated within commonly encountered locations replete with the sights and sounds of everyday life in the background. We show for the first time that sensing a body observed from the natural point of view as one's own during encoding enhances the accuracy with which narrative, visual, and contextual details (i.e., event details assessed by the cued recall questions) are retrieved both immediately after encoding and one week later, compared to well-matched control condition where ownership of the body in view is reduced.

In addition, we observed that memory for central details decreased between immediate and delayed testing points, while memory for peripheral details remained stable. Our finding was unexpected in light of previous research demonstrating that retrieving memories for film clips immediately after they have formed, as in the present study, preserves recall of both central and peripheral event details over time compared to when memories were not rehearsed directly following encoding (Sekeres et al., 2016). However, the discrepancy in results between the present study and that of Sekeres et al. (2016) highlights the importance of employing naturalistic paradigms that closely simulate real-life experiences when studying memory. Although Sekeres et al. (2016) presented a series of film clips on a computer screen, here we projected participants into a lifelike scene that felt more like visiting an actual location than watching a movie. By creating an immersive embodied experience within realistic scenes that more closely recreates naturalistic memory formation relative to viewing film clips, we show that peripheral information not pertinent to the main event narrative is low compared to central information immediately following memory formation. Because peripheral information is initially encoded worse than central information, there were fewer remembered details to forget compared to central event details, which may explain why memory declined for central but not peripheral details over time.

Turning to the subjective ratings, the present study also offers intriguing findings into the relationship between body ownership during the encoding of lifelike events and phenomenological properties of memory retrieval. First, we show that reliving of events experienced with a coherent sense of bodily self is higher immediately following encoding but fades over a week-long interval. Reliving is a hallmark of episodic memory that specifies wither remembering a past event incurs autonoetic consciousness, or the ability to mentally travel back in time and re-experience the event as if it were happening all over again (Tulving, 1985; Irish et al., 2011; Rubin et al., 2003). Reliving tends to be higher for recent compared to remote memories, and decreases significantly in the first week after memory formation alongside forgetting of specific episodic memory details (Tulving, 1985). Thus, the results of the present study suggest that although one's sense of body ownership during event encoding involves an initial increase in reliving relative to when body ownership is diminished, this effect is overshadowed by a more general reduction in reliving over time that applies to both memories encoded with and without feelings of body ownership. This is a key finding in relation to previous research which has not directly assessed the link between body ownership and reliving, and proposed effects after a delay of an hour (Bréchet et al., 2019, 2020) or a week (Bergouignan et al., 2014), rather than immediately after encoding. For example, recognition memory paradigms such as those employed by Bréchet et al. (2019, 2020) do not require reliving of the original event and can be performed purely on feelings of familiarity (Wheeler et al., 1997). Further, Bergouignan et al. (2014) use of remember/know judgments also do not provide a clear measure of reliving (Rubin and Umanath, 2015), but rather of overall memory strength (Wixted and Mickes, 2010) or belief in memory accuracy (Rubin et al., 2003; Rubin and Siegler, 2004; Wais et al., 2008). Thus, by directly asking participants to rate the degree to which they were able to relive the event during retrieval, we were able to show that body ownership during encoding immediately impacts reliving. Developing a basic understanding of how our capacity to relive the past relates to coherent feelings of body ownership during memory formation may prove important to the development of interventions designed to enhance reliving, which declines with normal aging (Levine et al., 2002; Piolino et al., 2006), following the onset of Alzheimer's Disease (Irish et al., 2011; Piolino et al., 2003), and in dissociative disorders such as depersonalization (Sierra and David, 2011). Although we were only able to initially enhance reliving, future research should investigate how these effects may be prolonged over longer time scales in clinical and healthy populations.

At delayed testing, memories encoded with intact feelings of body ownership were retrieved with higher vividness, emotional intensity, and belief in memory accuracy. Vividness and emotional intensity contribute to the overarching ability to relive past events (Irish et al., 2011). Thus, our results demonstrate that body ownership at encoding preserves two key memory components that enable reliving (i.e., vividness and emotional intensity), without impacting general reliving (i.e., the overall ability to experience a past event as if it were happening again, or as if one were mentally traveling back in time to when the event occurred) after a week-long delay. Further, our finding that vividness was reduced for events encoded without a coherent sense of body ownership at delayed retrieval is consistent with reports of reduced vividness for events encoded during an out-of-body illusion at a one-week follow up (Bergouignan et al., 2014). Together, the results of the present study and those of Bergouignan et al. (2014) demonstrate how memory vividness over time is determined by multiple aspects of bodily selfhood (i.e., body ownership, visual perspective, and perceived self-location), and that memory performance is optimal when all these aspects are aligned according to the natural spatial experience of self and world. In addition, we found that belief in memory accuracy (i.e., the degree to which an individual believes event details to be correct) is reduced for events encoded without a sense of ownership over the body in view after a week-long delay. Belief in memory accuracy is of significant practical importance as it separates memory for personal past events from imagination and is integral to reality monitoring processes (De Brigard, 2017; Johnson et al., 1988). False belief in events that have not actually occurred or failure to recall events that have occurred are characteristics of several psychiatric disorders including schizophrenia and dissociative disorders including depersonalization and derealization (APA, 2013). Our finding that lack of ownership of the body in view during encoding impairs belief in memory accuracy over time is important to understanding of these clinical disorders that not only involve misplaced belief in the accuracy of memories, but also disrupted bodily self-representation (Borda and Sass, 2015). In sum, the present results provide a comprehensive account of how a unified sense of body ownership at encoding supports the subjective re-experiencing of memories over time.

An important question posed by the current findings concerns the mechanism that explains the influence of body ownership on memory accuracy and re-experiencing.

We theorize that one's coherent sense of a multisensory body in the center of the spatial experience of self and world provides a fundamental egocentric “origo” for perceptual, affective, and cognitive processing; and that perturbing the coherence of this central own-body representation has widespread effects on self-representation and representation of the surroundings. Regarding episodic memory, a diminished sense of body ownership disrupts encoding processes, which in turn reduces the ability to accurately recall specific details of an event and re-experience it during retrieval. The hippocampus may be an important structure in determining how changes in multisensory self-coherence at encoding impact retrieval (Bergouignan et al., 2014), given its role in binding multifaceted streams of information together into a unified memory representation (Nyberg et al., 1996; Eichenbaum, 2000; Amaral et al., 2007). However, future research that measures neural activity as an event occurs is required to test whether feelings of body ownership affect hippocampal functioning at encoding, or manifest later during retrieval.

Another possible explanation for how own-body perception impacts memory accuracy and re-experiencing is that it increases the self-relevance of the event. In the present study, the feeling that the mannequin's body belonged to the participant in the synchronous condition could have meant that the unfolding scene was more self-relevant as the participant “was there, in the scene”, compared to when feelings of ownership over the mannequin were disrupted in the asynchronous condition as the scene was experienced as a “disembodied” stereoscopic movie. Here, we demonstrate that essential somatic experiences of body ownership, as opposed to the familiarity associated with experiencing a scene versus without one's visible body (Bréchet et al., 2019, 2020), enhances both the objective accuracy and subjective quality with which event details are re-experienced. A strength of our study design is that the mannequin's body was equally familiar in both the synchronous and asynchronous conditions, meaning that the critical difference between conditions was whether participants experienced a perceptual illusion of the mannequin's body as their own including somatic sensations originating from this artificial body. Consequently, the full-body ownership illusion may have increased the self-relevance of the event, which could have served as ‘integrative glue’ (Sui and Humphreys, 2015) that helped to bind different aspects of the scene together (i.e., narrative, visual, spatial, and kinaesthetic components). A rich tradition of memory research confirms that information tagged as self-relevant is better remembered (for review see Sui and Humphreys, 2015, 2017). For example, participants recall more episodic details about stimuli evaluated in relation to themselves compared to others (Cabeza and St. Jacques, 2007; Conway, 2005; Cunningham et al., 2008; Johnson et al., 2002; Lou et al., 2004; Rogers et al., 1977; Symons and Johnson, 1997; Turk et al., 2011) or semantic meaning (Craik et al., 1999; Leshikar et al., 2015). Self-reference also improves the ability to recall the context in which information is encoded (i.e., source memory; Cunningham et al., 2008; Sui and Humphreys, 2013), which is crucial to the reliving of past events.

In sum, the results of the present study emphasize the importance of the fundamental sense of one's own body to forming and retrieving memories for events. By manipulating the feeling of ownership over a mannequin's body through a perceptual illusion within lifelike scenes, we show that embodiment during memory formation is key to accurately retrieving episodic memory details and richly re-experiencing them over time. These findings can be used to develop new strategies aimed at improving the ability to vividly relive the past in the context of healthy aging and clinical disorders characterized by breakdowns in bodily selfhood and memory decline (e.g., dissociative disorders, Alzheimer's Disease).

### Limitations of the study

Interpretation of our findings is constrained by the present study's limitations. One limitation is that we did not control for potential differences in visuospatial attention or gaze patterns between conditions. For example, the mismatch between visual and tactile information in the asynchronous condition may have been more distracting for participants. Participants would then pay more attention to the mannequin's body at the expense of the rest of the scene, relative to the synchronous condition, which could explain the observed impairments in memory performance. However, we do not believe that increased attention to the mannequin's body explains the decreased memory accuracy and reliving in the asynchronous compared to synchronous condition as there were no differences in emotional intensity, vividness, or belief in memory accuracy immediately after encoding, which would be expected if attention were a confound.

Alternatively, the synchronous condition may have increased attention to the mannequin's body relative to the asynchronous condition, and distracted participants from the main scene. Yet we would then expect memory performance and re-experiencing to be worse in the synchronous condition, which was the opposite of our current findings. Moreover, previous research that has measured eye tracking within similar immersive videos did not find any differences in either gaze fixations between synchronous and asynchronous conditions or patterns of brain activity in the visual cortex that would be expected if one condition led to increased visual attention of the mannequin's body (Guterstam et al., 2015b; Iriye and Ehrsson, In Prep). Finally, with the exception for the relative timing of the tactile and visual stimuli, all other aspects of the experimental conditions were matched, and thus, we do not consider potential differences in visuospatial attention between the conditions to have significantly influenced our results.

## STAR★Methods

### Key resources table

**Table:** 

REAGENT or RESOURCE	SOURCE	IDENTIFIER
**Deposited data**		

Pseudo-anonymized source data from human subjects	Mendeley Data	https://doi.org/10.17632/b5rtnrdb6b.1

**Software and algorithms**		

RStudio Version 1.4.1717	R Studio Team (2021)	https://www.rstudio.com/
JASP Version 0.11.1	JASP Team (2019)	https://jasp-stats.org/
Audacity Version 2.3.2	Audacity Team (2018)	https://www.audacityteam.org/
Final Cut Pro Version 10.4.7	Apple Final Cut Pro X license and download [electronic resource]	https://www.apple.com/final-cut-pro/

### Resource availability

#### Lead contact

Further information and requests for resources should be directed to and will be fulfilled by the Lead Contact, Heather Iriye (heather.iriye@ki.se).

#### Materials availability

Participant instructions are listed in the Supplemental information file. Access to video stimuli is available from the Lead Contact upon request.

### Experimental model and subject details

Participants included 33 healthy young adults with no prior history of neurological or psychological impairment, and who were not taking medications that affected mood or cognitive functioning. Importantly, we only recruited participants who had not previously taken part in any experiments involving body illusions to ensure they would be naïve to the illusion induction. Three participants were excluded from the analysis as they did not return for the second session of the experiment. The final sample consisted of 30 participants (17 women, 13 men, mean age = 24.21, SD = 3.00). Participants provided written consent in a manner approved by the Swedish Ethical Review Authority. An a priori power analysis implemented in G∗Power 3.1.9.7 (Faul et al., 2007) was used to determine an appropriate sample size based on the results of Bréchet et al. (2019). The effect of exploring virtual scenes with, compared to without, a visible body on recognition memory accuracy was .48 (η^2^ = .19). The projected sample size to detect an effect of this magnitude with α = .05 was 28 (actual power = .96). Thus, a sample size of 30 is sufficiently large for the goals of our experiment.

### Method details

#### Materials

Stimuli consisted of 26 high-resolution digital 3D-videos depicting everyday life events in various famous locations around Stockholm, Sweden. We chose recognizable local places of interest to recreate natural real-life experiences for participants and enhance the ecological validity of the study. Videos were presented through an Oculus Rift DK2 head mounted display (HMD) unit. Each video included a stereoscopic view of a mannequin’s body seen from a supine, first-person perspective. Stereoscopic vision was achieved by filming each event with two GoPro Hero 7 cameras mounted side-by-side on a tripod to mimic left and right eye positions. The recordings from the left and right cameras were placed next to one another on the HMD screen (1920 x 1080 pixels) such that footage from the left camera was sent to the left half of the screen and footage from the right camera was sent to the right half of the screen. Throughout each video, a white Styrofoam ball (6.5 cm in diameter) attached to a wooden stick (1 m long) repeatedly stroked the mannequin’s torso from the sternum to the belly button in a downwards direction every 2 seconds for 40 seconds total (O'Kane and Ehrsson, 2021). Each stroke lasted approximately 1 second. After 20 seconds, an everyday scene unfolded in the video (i.e., the two main actors engage in a short conversation based on a unique, visible item (see further below). The video footage of the mannequin being stroked by the wooden stick was filmed separately against a green screen and imposed onto the footage of the everyday scenes using Final Cut Pro X 10.4.7. An audio track of the dialog was recorded separately from the video in a soundproof environment using a Rode Videomic Go microphone. Background noises appropriate to each video (e.g. birds, wind, distant conversations, traffic) were layered underneath the dialog using Audacity® 2.3.2 and the final audio track was integrated with its respective video using Final Cut Pro X 10.4.7. A second audio track only heard by the experimenter was added to each video to cue precise timing and duration of the tactile stimuli. Audio tracks delivered to the experimenter and participant were separated by an Edirol USB AudioCapture sound card (model number: UA-25EX, 24-bit, 96 kHz).

To ensure that the observed effects of body ownership on memory accuracy and subjective reliving would not be due to differences in the complexity of the individual videos, two independent raters judged each video in terms of multiple sub-categories of complexity and composite scores of each sub-category were used to divide videos into two groups (see Table S1) to minimize differences in average complexity between the groups (see Table S2; Bonasia et al., 2018; Sekeres et al., 2016). Assignment of each video group to an experimental condition was counterbalanced across participants. The independent raters judged each video in terms of its visual, audio, narrative, and emotional complexity, using five-point Likert scales (1 = Low, Five = High). Visual complexity was measured along three dimensions concerning complexity of the background scene, amount of movement, and number of characters. Auditory complexity referred to complexity of the background audio and narrative complexity referred to complexity of the storyline. Emotional complexity was characterized according to degree of sadness, excitement, joy, anger, disgust, fear, and shame present within each video. A two-way random effects model was run for each complexity dimension to assess inter-rater reliability. The emotional sub-categories of sadness, anger, disgust, fear, and shame did not display enough variance to run the analysis as all ratings for both raters were at floor level (i.e. one). The average intraclass correlation coefficient across complexity categories was 0.78 (*SD* = .08), demonstrating a reliable degree of agreement between raters (see Table S3 for means from each sub-category).

#### Procedure

The study was comprised of two sessions spaced one week apart. During the first session, participants were fitted with the HMD and repeatedly watched the 24 videos while lying in a reclined position, such that the location of the participants’ real bodies was aligned with a first-person view of the mannequin’s body in the video (see Figures 1 and S1). Each video was associated with a unique title that was presented in the centre of the screen for 2.5 seconds before the video began and visible at the top of the screen as it was playing. To induce an illusory feeling of ownership over the mannequin in half of the videos, participants felt touches on their torso at the same time that they saw the mannequin’s torso being touched in the video (i.e. synchronous visuotactile stimulation; e.g. Petkova and Ehrsson, 2008). As a control condition, participants saw and felt touches in an alternating pattern in the other half of the videos, i.e. asynchronous visuotactile stimulation, which is known to significantly reduce or eliminate the illusion (Petkova and Ehrsson, 2008). Critically, the strokes in the asynchronous condition were identical in magnitude to those in the synchronous condition, and the only difference between conditions was the relative timing of seen and felt strokes. Participants were instructed to remember as much as possible about each video, including the title. Videos were separated by a random jittered fixation cross placed in the centre of the field of view that was visible for a period of 2.5–10 seconds (4 x 2.5s, 3 x 5s, 2 x 7s, 1 x 10s). Participants viewed individual videos a total of three times over the course of nine runs, each consisting of eight videos. Videos were repeated three times to ensure that participants would be able to recall each video one week later during session two, which was determined by pilot testing. Participants were given a five-minute break at the end of every third run to keep them engaged in the task. Video presentation was pseudo-randomized across runs, such that no condition was repeated more than twice in a row.

Immediately after participants finished watching the videos, participants completed a cued recall test comprised of five questions per video to assess objective memory accuracy. Three questions were related to the main storyline (e.g. Which animal in the museum was Heather surprised to see?) and two questions concerned background peripheral details not important to the storyline (e.g. How many scooters were parked outside the museum?). To assess memory phenomenology, participants rated reliving, emotional intensity, vividness, and degree of belief in memory accuracy on seven-point Likert scales (1 = None, 7 = High; see Methods S1 for instructions to participants; Iriye and St. Jacques, 2021). Cued recall questions and subjective ratings were completed on a computer. One week later, participants returned to the lab for a second session where they completed a cued recall accuracy test with new questions and the same subjective ratings made during session one. Cued recall questions were randomly assigned to either session one or session two for each participant and presented in random order.

We also measured the strength of the full body illusion during session two, directly before administering the cued recall test. Participants were immersed within two previously unseen videos, one with synchronous and the other with asynchronous visuotactile stimulation. These new videos were shown purely to test the strength of the full body illusion and were not related to the cued recall test. After viewing each video, participants were asked to rate three statements pertaining to degree of embodiment over the mannequin and three control statements on seven-point Likert scales (−3 = Strongly Disagree, 0 = Neutral, 3 = Strongly Agree; see Table 1) presented in random order on a computer screen. S1 and S2 assessed referral of touch from the participant’s actual body to the mannequin indicating coherent multisensory perception of the body and visuotactile binding, while S3 assessed degree of explicitly perceived ownership over the mannequin’s body (Petkova and Ehrsson, 2008). To assess the overall strength of the full-body illusion we calculated the average score of statements S1, S2 and S3 in line with earlier studies (van der Hoort and Ehrsson, 2014; Guterstam et al., 2015a, 2015b; Preuss and Ehrsson, 2019). Three additional control statements were included (S4, S5 and S6) to control for suggestibility, response bias and confabulation. Finally, we asked participants to rate the degree of familiarity with the physical locations depicted in the videos on 7-point Likert scales (1 = I visited the location once, 7 =I visited the locations more times than I can count) for an analysis outside of the scope of the present study. We did not collect threat-evoked SCR as objective evidence of the current full-body illusion as many previous studies have already done so (Ehrsson, 2007; Guterstam et al., 2015a, 2015b; Petkova and Ehrsson, 2008; Tacikowski et al., 2020) and established the validity of the current synchronous versus asynchronous manipulation. More importantly, we did not want emotionally salient threat experiences to influence memory encoding.

### Quantification and statistical analysis

We calculated illusion statement ratings corrected for suggestibility and response bias by subtracting average control statement ratings from average illusion statement ratings separately for each condition. Cued recall questions were coded for accuracy using strict criteria in which responses had to exactly match the correct response to be scored as correct (e.g. *What type of injury was Heather recovering from?* Correct answer: *A sprained ankle*, Incorrect Answer: *A foot injury*). Using a more lenient criteria that allowed for partial marks did not change the overall pattern of the results. The percentage of correct responses for central and peripheral details for both conditions and testing points was calculated for each participant. Subjective ratings were averaged across videos to create a mean score for each rating. Shapiro-Wilk tests were used to assess normality of the data from the illusion induction questionnaire, cued recall test, and subjective ratings. We used repeated measures ANOVAs to analyse data that was normally distributed and Wilcoxon signed-rank tests to assess data that was not normally distributed. Follow-up t-tests were corrected for multiple comparisons using Bonferroni corrections. Greenhouse Geisser corrections were applied where the data violated assumptions of sphericity. Significance was defined as *p* < .05. Post-hoc Spearman’s rank-order correlations determined the relationship between the strength of the full-body ownership illusion (i.e. average illusion – average control statement ratings in the synchronous condition), average cued recall accuracy, and average individual subjective ratings in the synchronous condition. We chose the Spearman correlation as the data was not normally distributed. All statistical tests were conducted using JASP Team (2019).

## Data Availability

Pseudo-anonymized source data (questionnaire ratings, cued recall accuracy, and subjective ratings) has been deposited at Mendeley and are publicly available as of the date of publication. Accession numbers are listed in the key resources table. Any additional information required to reanalyze the data reported in this paper is available from the Lead Contact upon request.

## Footnotes

Supplemental information can be found online at https://doi.org/10.1016/j.isci.2021.103584.
