# Supplementary material for: Perceptual illusion of body-ownership within an immersive realistic environment enhances memory accuracy and re-experiencing
Source: iScience. 2021 Dec 8;25(1):103584. doi: 10.1016/j.isci.2021.103584 (PMC8717413; doi:10.1016/j.isci.2021.103584)
Supplement: Document S1. Figures S1 and S2, Tables S1–S3, and Methods S1 [file mmc1.pdf]

iScience, Volume *25*

Supplemental information

Perceptual illusion of body-ownership within an immersive realistic environment enhances memory accuracy and re-experiencing

Heather Iriye and H. Henrik Ehrsson

SUPPLEMENTAL INFORMATION

SUPPLEMENTAL FIGURES

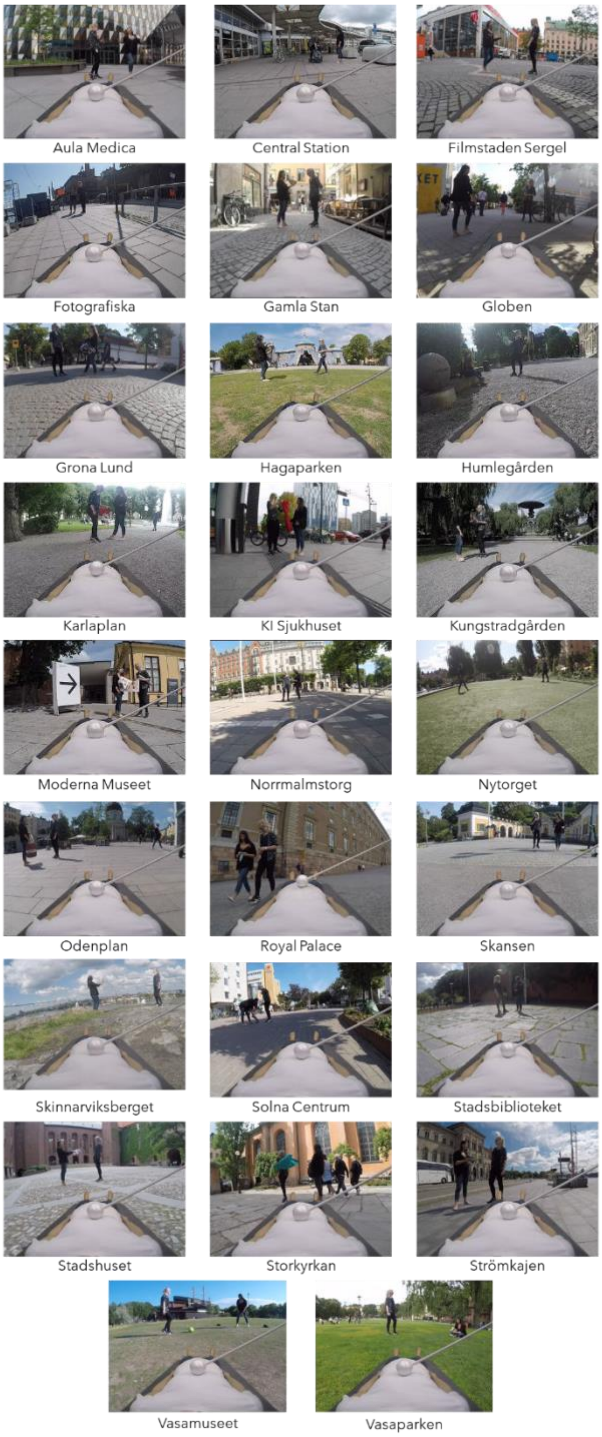

Figure S1. Video Stimuli, Related to STAR Methods. Screenshots from each of the 26 immersive videos depicting famous locations around Stockholm, Sweden.

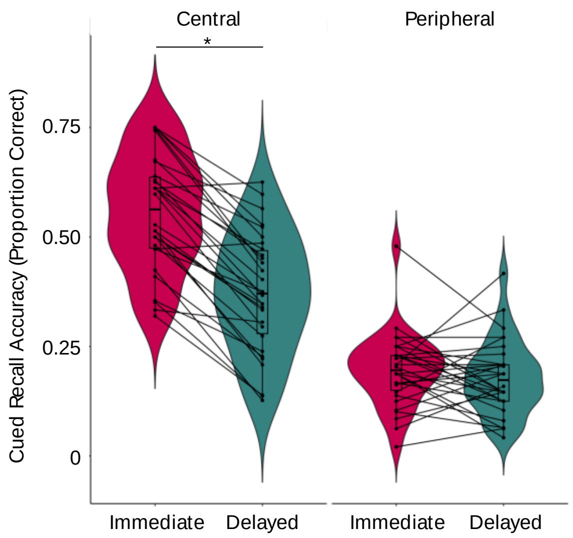

Figure S2. Interaction Between Testing Point and Detail Type on Cued Recall Accuracy, Related to Figure 3. Average cued recall accuracy per participant according to testing point and detail type. Retrieval of central details decreased between immediate and delayed testing points, whereas retrieval of peripheral details did not change over time.

SUPPLEMENTAL TABLES

Table S1.
Average Complexity Scores Per Sub-Category According to Group Assignment, Related to STAR Methods

Video Title	**Visual**	**Auditory**	**Narrative**	**Emotional**	**Group**
Moderna Museet	2.00	2.50	1.50	1.36	1
Fotografiska	1.83	1.00	2.00	1.93	1
Storkyrkan	2.83	3.00	2.00	1.00	1
Solna Centrum	2.50	1.50	2.00	1.29	1
Vasa Museet	2.67	2.50	2.00	1.21	1
Gamla Stan	3.50	3.00	2.50	1.64	1
Kungstradgården	2.00	2.00	2.50	1.00	1
Stadshuset	2.33	3.00	2.50	1.14	1
Hagaparken	2.67	1.00	2.50	1.14	1
Odenplan	2.83	4.00	3.00	1.07	1
Karlaplan	2.33	1.50	3.00	1.07	1
Filmstaden Sergel	2.67	3.00	3.50	1.21	1
Central Station	3.00	2.00	1.00	1.93	2
KI Sjukhuset	4.50	2.50	1.50	2.43	2
Humlegården	1.67	1.50	2.00	1.07	2
Royal Palace	2.17	3.00	2.00	1.00	2
Strömkajen	2.50	2.00	2.00	1.29	2
Skansen	2.33	1.50	2.50	1.14	2
Stadsbiblioteket	1.50	1.00	2.50	1.43	2
Skinnarviksberget	1.83	3.50	2.50	1.21	2
Nytorget	2.33	3.00	2.50	1.07	2
Vasaparken	2.33	2.50	2.50	1.00	2
Globen	3.00	3.00	3.50	1.14	2
Grona Lund	2.83	3.00	3.50	1.14	2

Table S2.
Difference in Average Complexity Between Groups of Video Stimuli, Related to STAR Methods

	Complexity Category			
	**Visual**	**Auditory**	**Narrative**	**Emotional**
Group 1	2.51	2.33	2.42	1.26
Group 2	2.00	2.46	2.33	1.32
**Difference**	0.01	-0.13	-0.08	-0.07

Table S3.
Average Intraclass Correlation Coefficients of Complexity Sub-Categories, Related to STAR Methods

**Visual Complexity Measure**	**Intraclass Correlation Coefficient**
Background	0.80
Movement	0.73
Number of Characters	0.82
**Auditory Complexity Measure**	
Background Audio	0.88
**Narrative Complexity Measure**	
Storyline	0.62
**Emotional Complexity**	
Excitement	0.81
Joy	0.77
Anger	Not enough variance
Sadness	Not enough variance
Disgust	Not enough variance
Fear	Not enough variance
Shame	Not enough variance

METHODS S1

Participant Instructions: Cued Recall & Subjective Ratings, Related to STAR Methods

You will be asked to answer five questions about details within the video clip (e.g., what color was the dog?). Additionally, you will be asked to subjectively rate your memory for each video clip on several properties:

1. Emotional Intensity: Emotions can be felt with different intensities. For example, a negative memory can be extremely sad while another may be just somewhat sad.

Similarly, a positive memory can be really exciting and euphoric and another can be calm and relaxing. Regardless of whether the memory was positive or negative, how intense was the emotion?

As I remember the (video clip), the emotional intensity I feel is:

1 2 3 4 5 6 7

None Minimal Moderate High

2. Reliving: This question relates to a feeling of experiencing the event again as if it were happening right now, or as if you were mentally traveling back in time to when the event occurred. How much do you feel that you can relive the memory?

As I remember the (video clip), the degree of reliving I feel is:

1 2 3 4 5 6 7

None Minimal Moderate High

3. Vividness: This question refers to the clarity with which you can see the event in your mind. The visual resolution of some memories may be as clear as if watching a high-definition show in front of you, whereas the visual resolution of other memories may be much poorer.

As I remember the (video clip), the vividness of my recollection is:

1 2 3 4 5 6 7

None Minimal Moderate High

4. Belief in memory accuracy: This question refers to how strongly you believe that your memory of the event in the video clip is an accurate representation of what actually happened. We

may be highly confident in the accuracy of some memories, while not at all confident in the accuracy of others.

As I remember the (video clip), I feel the accuracy of my memory is:

1	2	3	4	5	6	7
None		Minimal		Moderate		High
